# The relationship between maximal expiratory pressure values and critical outcomes in mechanically ventilated patients: a post hoc analysis of an observational study

**DOI:** 10.1186/s13613-020-00791-4

**Published:** 2021-01-13

**Authors:** Yann Combret, Guillaume Prieur, Roger Hilfiker, Francis-Edouard Gravier, Pauline Smondack, Olivier Contal, Bouchra Lamia, Tristan Bonnevie, Clément Medrinal

**Affiliations:** 1 Intensive Care Unit, Le Havre Hospital, 76600 Le Havre, France; 2 Research and Clinical Experimentation Institute (IREC), Pulmonology, ORL and Dermatology, Louvain Catholic University, 1200 Brussels, Belgium; 3 Institute for Research and Innovation in Biomedicine (IRIB), Normandie University, UNIROUEN, UPRES EA3830-GRHV, 76000 Rouen, France; 4 School of Health Sciences, University of Applied Sciences and Arts Western Switzerland Valais (HES-SO Valais-Wallis), Leukerbad, Switzerland; 5 ADIR Association, Rouen University Hospital, 76000 Rouen, France; 6 University of Applied Sciences and Arts, Western Switzerland (HES-SO), Lausanne, Switzerland; 7 Pulmonology Department, Le Havre Hospital, 76600 Le Havre, France; 8 Intensive Care Unit, Respiratory Department, Rouen University Hospital, Rouen, France; 9 Paris-Saclay University, UVSQ, Erphan, 78000 Versailles, France; 10 Saint Michel School of Physiotherapy, 75015 Paris, France

**Keywords:** Maximal expiratory pressure, Extubation failure, Mechanical ventilation, Intensive care unit

## Abstract

**Background:**

Little interest has been paid to expiratory muscle strength, and the impact of expiratory muscle weakness on critical outcomes is not known. Very few studies assessed the relationship between maximal expiratory pressure (MEP) and critical outcomes. The aim of this study was to investigate the relationship between MEP and critical outcomes.

**Methods:**

This work was a secondary analysis of a prospective, observational study of adult patients who required mechanical ventilation for ≥ 24 h in an 18-bed ICU. MEP was assessed before extubation after a successful, spontaneous breathing trial. The relationships between MEP and extubation failure, and short-term (30 days) mortality, were investigated. Univariate logistic regressions were computed to investigate the relationship between MEP values and critical outcomes. Two multivariate analyses, with and without maximal inspiratory pressure (MIP), both adjusted using principal component analysis, were undertaken. Unadjusted and adjusted ROC curves were computed to compare the respective ability of MEP, MIP and the combination of both measures to discriminate patients with and without extubation failure or premature death.

**Results:**

One hundred and twenty-four patients were included. Median age was 66 years (IQR 18) and median mechanical ventilation duration was 7 days (IQR 6). Extubation failure rate was 15% (18/124 patients) and the rate for 30-day mortality was 11% (14/124 patient). Higher MEP values were significantly associated with a lower risk of extubation failure in the univariate analysis [OR 0.96 95% CI (0.93–0.98)], but not with short-term mortality. MEP was independently linked with extubation failure when MIP was not included in the multivariate model, but not when it was included, despite limited collinearity between these variables. This study was not able to differentiate the respective abilities of MEP, MIP, and their combination to discriminate patients with extubation failure or premature death (adjusted AUC for the combination of MEP and MIP: 0.825 and 0.650 for extubation failure and premature death, respectively).

**Conclusions:**

MEP is related to extubation failure. But, the results did not support its use as a substitute for MIP, since the relationship between MEP and critical outcomes was no longer significant when MIP was included. The use of MIP and MEP measurements combined did not reach higher discriminative capacities for critical outcomes that MEP or MIP alone.

*Trial Registration* This study was retrospectively registered at https://clinicaltrials.gov/ct2/show/NCT02363231?cond=NCT02363231&draw=2&rank=1 (NCT02363231) in 13 February 2015

## Background

Mechanical ventilation (MV) is well known to cause rapid, severe, respiratory muscle dysfunction and weakness [1, 2]. Respiratory muscle weakness has been linked to poor outcomes, including ventilator-weaning failure, extubation failure and death [3–6]. Research into the impact of inspiratory muscle weakness on critical outcomes has focussed on diaphragm ultrasonography and maximal inspiratory pressure (MIP) measurements [6, 7]. In contrast, few authors have examined the impact of expiratory muscle strength on critical outcomes, despite the fact that expiratory muscles participate in respiratory system homeostasis, cough capacity, facilitate diaphragmatic contractile efficiency and reduce hyperinflation, especially in patients with impeding respiratory failure [8–10].

Studies into expiratory muscle strength frequently measure maximal expiratory pressure (MEP), which was recently shown to be reliable in both intubated and non-intubated patients, and strongly correlated with MIP in both conditions [11–13]. MEP values at the time of weaning are generally lower than predicted values; it has also been reported that MEP is reduced in patients with failed extubation, compared to those who were successfully extubated [11, 14].

Despite this, the clinical significance of a low MEP on critical outcomes is currently unknown. The primary objective of this study, therefore, was to explore the relationship between MEP values and extubation failure, and short-term mortality. Secondary objectives were (1) to explore the impact of MEP values on critical outcomes whether MIP was included or not and; (2) to compare the ability of MEP and MIP, separately and combined, to discriminate patients with extubation failure and premature death.

## Methods

### Study design and setting

This study was a secondary analysis of an observational study carried out between January and December 2014 in an 18-bed medical intensive care unit (ICU) [7]. The present study was authorised by the Comité de Protection des Personnes Nord-Ouest III, conducted according to the Declaration of Helsinki and registered on clinicaltrials.gov (NCT02363231). All patients participated voluntarily. The study is reported according to the Strengthening the Reporting of Observational Studies in Epidemiology (STROBE) statement.

### Participants

The same inclusion criteria were used for the secondary analysis as for the original study [7]. Briefly, participants were adults (≥ 18 years) hospitalised in ICU who had been on MV for ≥ 24 h and had undergone a successful spontaneous breathing trial (SBT). The exclusion criteria have been detailed elsewhere [7]. Briefly, they included an inability to undergo MEP measurement, a decision to withhold treatments or previously documented respiratory muscle weakness.

### Study procedure

Following cessation of all sedatives, patients’ states of arousal were assessed several times per day. Once alert, cooperative (Ramsay score at 2) and responsive to simple orders, a SBT was undertaken using pressure support (inspiratory positive airway pressure: 7cmH_2_O and expiratory positive airway pressure: 0cmH_2_O) for 30 to 120 min [15]. If the SBT was successful, extubation was planned and patient eligibility was assessed. Expiratory muscle strength was assessed following inclusion by measurement of MEP [12, 16].

### Measurement of MEP and outcome collection

MEP was measured using an electronic manometer (MicroRPM, Eolys) with a unidirectional valve connected to the endotracheal intubation tube with a catheter mount. The procedure is fully described elsewhere [7]. Three measurements were made, all with the patient disconnected from the ventilator, and the best value was recorded. MIP was also measured according to the Marini method [16]. Demographic data, reason for admission, comorbidities and other factors associated with the ICU stay were also collected.

### Study outcomes

Extubation failure (defined as reintubation within 48 h) and short-term mortality (i.e. at 30 days) were recorded. Due to the observational design, reintubation indications were not protocolised in the present study, and were left to and made by the attending clinician, regardless of the study protocol. Nonetheless, reintubation indications in our ICU department encompasses respiratory failure, hypoxemia, laryngeal oedema, inability to ensure airway protection, shock, decreased level of consciousness, copious secretions that could not be cleared despite adequate treatments.

### Statistical methods

Patients characteristics are reported as numbers (and/or percentages) for categorical data and as medians (interquartile ranges) for continuous data. We calculated univariable logistic regressions for the dependent variables extubation failure and mortality, including the independent variables age, sex, comorbidities, ICU-acquired weakness (ICU-Aw), SAPS II score, days of ventilator use (prior to test day), days of neuromuscular blocker use, as well as for MEP and MIP. We reported Odds Ratio with 95% confidence intervals as well as standardised odds ratios. For the standardisation, the continuous independent variables were divided by two standard deviations of the variables [17]. This allows the comparison of odds ratio across variables and to compare them also to odds ratio from binary independent variables [17]. Because the number of events did not allow for the adjustment with all relevant potential confounders, we used a method proposed by Riley et al. where a principal component analysis was performed including a set of predefined predictors, and the first component was used in the multivariable adjusted logistic regressions for MEP [18]. The same model was then computed, but with MIP included to explore the impact of the latter on the relationship between MEP and critical outcomes, after collinearity was verified. We calculated receiver operating characteristics (ROC) curves and area under the ROC curves (AUC) for the variables MEP, MIP, and the combination of MEP and MIP, for both outcomes extubation failure and mortality, unadjusted and adjusted for the principal component score. The null-hypothesis of equality of the AUCs for the different predictors was tested with a method proposed by DeLong and Clarke-Pearson (implement in the command *roccomp* in Stata version 16.1) [19]. This test produces a p-value from a Chi-squared test. We used Stata version 16.1. (StataCorp, Texas, USA) for all calculations and a *p *value < 0.05 was considered significant.

## Results

Among the 856 patients screened for the original 2014 observational study, 186 were eligible and 124 were finally included (main exclusion reasons: delirium and impossibility of measuring MEP) [7]. Patients characteristics are described in Table 1. The complete flow diagram of patient inclusions can be found elsewhere [7].

**Table 1.** Patients characteristics

	*N* = 124
Sex F/M, *n* Age, median (IQR) Body mass index (kg/m²), median (IQR) SAPS II at ICU admission, median (IQR) ICU admission in the last years, *n* (%)	51/73 66 (18) 27.8 (6.6) 45 (24) 8 (6)
Main diagnosis	
Pneumonia, *n* (%) Sepsis, *n* (%) COPD/asthma exacerbation, *n* (%) Cardiac failure, *n* (%) Drug overdose/acute mental status change, *n* (%) Intra-abdominal sepsis with surgery, *n* (%) Trauma, *n* (%)	33 (27) 9 (7) 21 (17) 19 (15) 16 (13) 20 (16) 6 (5)
Comorbidities	
Chronic pulmonary disease, *n* (%) Obesity, *n* (%) Chronic cardiac insufficiency, *n* (%) Chronic kidney disease, *n* (%) Cancer, *n* (%) Diabetes, *n* (%) Chronic alcoholism, n (%) Active smoking, *n* (%) Malnourishment, *n* (%)	28 (22) 36 (29) 19 (15) 20 (16) 19 (15) 30 (24) 30 (24) 29 (23) 10 (8)
Between admission and extubation	
Septic shock, *n* (%) ARDS, *n* (%) Renal failure, *n* (%) Use of catecholamines, *n* (%) Use of neuromuscular blockers, *n* (%) No. of days of neuromuscular blockers, median (IQR) Use of corticosteroids, *n* (%) ICU length of stay, median (IQR) Ventilator use (days), median (IQR) MIP (cmH_2_O), median (IQR) MEP (cmH_2_O), median (IQR) ICU-acquired weakness, *n* (%)^a^	61 (49) 11 (9) 38 (31) 64 (52) 76 (61) 1 (2) 34 (27) 10 (9) 7 (6) 47 (44) 47 (24) 49 (49)

*ARDS* acute respiratory distress syndrome, *COPD* chronic obstructive pulmonary disease, *ICU* intensive care unit, *IQR* interquartile range, *MEP* maximal expiratory pressure, *SAPS* Simplified Acute Physiology Score

^a^99 of the 124 patients were assessed for ICU-acquired weakness (MRC score)

In the univariate logistic regression analyses, MEP, MIP, body mass index (BMI), neuromuscular blocker administration duration and MV duration were significantly related to extubation failure. Conversely, neither MEP nor MIP was associated with short-term mortality; and age, SAPS II and BMI were the only variables significantly related with mortality at 30 days (Table 2).

**Table 2.** Univariate logistic regression analyses for critical outcomes

	Extubation failure			Death at 30 days		
	OR (95% CI)	Std OR (95% CI)	*p* value	OR (95% CI)	Std OR (95% CI)	*p* value
Age	0.98 (0.95–1.02)	0.71 (0.27–1.89)	0.50	1.04 (1–1.08)	3.51 (1.18–10.45)	0.02
Sex	0.89 (0.32–2.49)	0.89 (0.32–2.49)	0.83	0.61 (0.24–1.55)	0.61 (0.24–1.55)	0.32
BMI	0.90 (0.81–0.98)	0.22 (0.05–0.86)	0.03	0.87 (0.79–0.95)	0.14 (0.03–0.50)	0.003
Neuromuscular blocker administration duration	1.36 (1.11–1.67)	3.85 (1.57–9.43)	0.003	0.97 (0.79–1.19)	0.89 (0.36–2.2)	0.80
MV duration	1.16 (1.05–1.28)	4.26 (1.61–11.25)	0.003	1.05 (0.96–1.14)	1.66 (0.73–3.78	0.22
SAPS II	0.99 (0.96–1.02)	0.64 (0.22–1.85)	0.41	1.02 (1.00–1.05)	2.41 (1.01–5.74)	0.046
Cancer	0.29 (0.04–2.30)	0.29 (0.04–2.30)	0.24	2.09 (0.70–6.20)	2.09 (0.70–6.20)	0.18
Chronic cardiac insufficiency	1.13 (0.29–4.33)	1.13 (0.29–4.33)	0.86	2.82 (0.97–8.15)	2.82 (0.97–8.15)	0.056
Chronic kidney disease	1.05 (0.27–4.01)	1.05 (0.27–4.01)	0.95	0.39 (0.08–1.81)	0.39 (0.08–1.81)	0.23
Chronic pulmonary disease	0.17 (0.02–1.35)	0.17 (0.02–1.35)	0.095	0.83 (0.28–2.45)	0.83 (0.28–2.45)	0.73
Diabetes	0.59 (0.16–2.18)	0.59 (0.16–2.18)	0.42	1.29 (0.48–3.46)	1.29 (0.48–3.46)	0.62
Obesity	0.66 (0.20–2.16)	0.66 (0.20–2.16)	0.49	0.73 (0.26–2.00)	0.73 (0.26–2.00)	0.54
Chronic alcoholism	0.35 (0.08–1.61)	0.35 (0.08–1.61)	0.18	0.53 (0.17–1.70)	0.53 (0.17–1.70)	0.29
Active smoking	0.62 (0.17–2.29)	0.62 (0.17–2.29)	0.47	1.36 (0.50–3.68)	1.36 (0.50–3.68)	0.54
ICU-acquired weakness	2.95 (0.86–10.14)	2.95 (0.86–10.14)	0.086	1.99 (0.71–5.58)	1.99 (0.71–5.58)	0.19
Malnourishment	0.63 (0.08–5.33)	0.63 (0.08–5.33)	0.68	2.95 (0.76–11.40)	2.95 (0.76–11.40)	0.12
MEP	0.96 (0.93–0.98)	0.06 (0.01–0.44)	0.005	0.99 (0.98–1)	0.72 (0.28–1.88)	0.51
MIP	0.90 (0.84–0.96)	0.02 (0.001–0.21)	0.001	0.97 (0.94–1)	0.39 (0.13–1.16)	0.09

*OR* odds ratios, *Std OR* standardised odds ratio, *CI* confidence interval, *BMI* body mass index, *MV* mechanical ventilation, *MIP* maximal inspiratory pressure, *MEP* maximal expiratory pressure

In the multivariate logistic regression models, MEP was significantly associated with extubation failure in the primary model without MIP [OR 0.96 95% CI (0.94–0.99); *p* = 0.01], but was not in the model that included MIP [OR 0.99 95% CI (0.96–1.02); *p* = 0.50] (Table 3). MEP was not significantly associated with mortality at 30 days in the multivariate analysis model, whether MIP was included in the model or not (Table 3). Collinearity diagnostics of MEP and MIP revealed very limited collinearity between both measures with a variance inflation factor (VIF) of 1.83 (square root VIF = 1.35).

**Table 3.** Multivariate logistic regression models for MEP associations with critical outcomes: with and without MIP

	Extubation failure			Death at 30 days		
	OR	95% CI	*p* value	OR	95% CI	*p* value
Model without MIP						
MEP	0.97	(0.94–0.99)	0.013	0.99	(0.98–1)	0.53
Principal component 1 score	0.59	(0.40–0.89)	0.011	1.04	(0.75–1.4)	0.81
Model with MIP						
MEP	0.99	(0.96–1.02)	0.50	1	(0.98–1.02	0.46
MIP	0.92	(0.86–0.99)	0.019	0.97	(0.93–1)	0.08
Principal component 1 score	0.61	(0.40–0.92)	0.019	1.03	(0.74–1.42)	0.86

*OR* odds ratio, *CI* confidence interval, *MIP* maximal inspiratory pressure, *MEP* maximal expiratory pressure

**Fig. 1:**
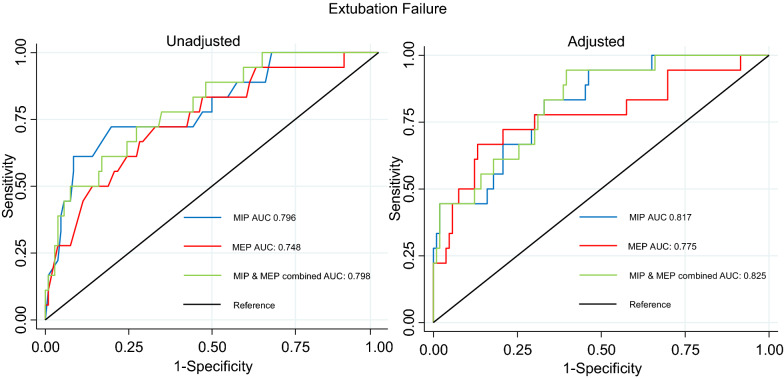
ROC curves representing MEP and MIP (separated and combined) abilities to discriminate patients with extubation failure

**Fig. 2:**
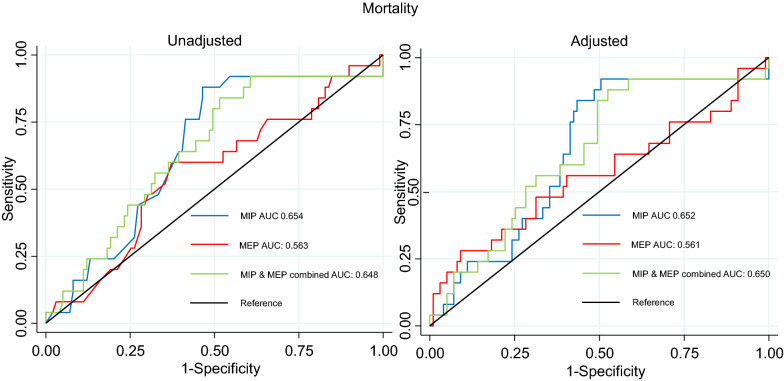
ROC curves representing MEP and MIP (separated and combined) abilities to discriminate patients with premature death

Finally, AUC (adjusted and unadjusted) were not significantly different for extubation failure between MEP, MIP and the combination of MEP and MIP (unadjusted AUC: 0.748 vs 0.796 and 0.798, respectively, *p* = 0.24; adjusted AUC: 0.775 vs 0.817 and 0.825, respectively, *p* = 0.14) (Fig. 1). Similarly, AUC were not different for early mortality between MEP, MIP and the combination of MEP and MIP (unadjusted AUC: 0.563 vs 0.654 and 0.648, respectively, *p* = 0.10; adjusted AUC: 0.561 vs 0.652 and 0.650, respectively, *p* = 0.23) (Fig. 2).

## Discussion

The results of this study revealed that (1) higher MEP values were associated with a lower risk of extubation failure in the primary univariate logistic regression; (2) in the multivariate logistic regression models, higher MEP values were associated with reduced extubation failure in the model without MIP, but not in the model that included MIP; (3) MEP values were not associated with short-term mortality in the univariate or multivariate analysis; (4) adjusted and unadjusted AUC for MEP, MIP and the combination of MEP and MIP similarly discriminated patients with extubation failure and short-term mortality.

The present results showed that higher MEP values were associated with a lower risk of extubation failure. The primary reason for this result could be the key role of the expiratory muscles in generating a sufficiently effective cough to ensure airway clearance following extubation [20]. Indeed, previous work based on a multivariate analysis adjusted for MV duration, the presence of chronic respiratory failure and ICU-Aw indicated that higher MEP values are associated with a reduction of extubation failure [OR 0.98 95% CI (0.97–1.0); *p* = 0.04] [9]. Furthermore, in that study by Terzi et al., patients who required respiratory support, non-invasive ventilation (NIV) due to respiratory distress, or mechanical cough assistance due to an inability to clear secretions also had lower MEP values than those who underwent simple extubation (30 vs 53 cmH_2_O) [9]. Indeed, ineffective cough is a factor associated with extubation failure, even though MEP is not the only factor contributing to cough efficiency [21, 22]. Noteworthy, cough was not directly measured in the present study, and the use of mechanical cough assistance after extubation was not reported. The use of such devices was, however, based on clinicians’ decisions and was not influenced by the measures undertaken for the present study.

One hypothesis behind the present analysis was that the expiratory muscles contribute to maintaining the balance between respiratory load and respiratory system capacity by enhancing the activity of the inspiratory muscles [8]. Two multivariate regression analysis models were then computed, with and without MIP, to explore the relationship between MEP and critical outcomes, when MIP was also taken into account. Since MEP was related to extubation failure in the model without MIP, but not in the model including MIP, expiratory muscles weakness did not occur in isolation from inspiratory muscle weakness in the present study. One explanation for this could be that two of the variables related to extubation failure in the univariate analysis (namely MV duration and neuromuscular blocker administration duration) have previously been linked to both inspiratory and expiratory muscle weakness [9, 23, 24]. In a previous study by Terzi et al., patients who required mechanical cough assistance (i.e. patients with expiratory muscle weakness) had a higher MV duration compared to those who required NIV (19.8 vs 17.4 days, respectively; *p* = 0.02) [9]. Expiratory muscle strength could therefore decrease at a later stage than inspiratory muscle strength, explaining the lack of relationship between MEP and critical outcomes when MIP is also included in the model.

MEP is an easy-to-use bedside tool that can easily and quickly be measured by clinicians. It is, however, worthy of note that one disadvantage of MEP evaluation is that the patient must be sufficiently aroused to voluntarily generate a maximal effort. Another key point is that, contrary to MIP, MEP cannot be directly measured on modern ventilator machines without disconnecting the patient from the ventilator. According to the AUC results, we were unable in the present study to differentiate the respective abilities of MEP and MIP separated or combined to discriminate premature death, but those three AUCs were low and unlikely to provide clinical guidance for that outcome. AUCs for the discrimination of extubation failure were higher than for short-term death, but we were unable to isolate any difference between each of the measurements undertaken. One could then consider than using MIP measurement alone could lead to similar results and would be quicker than the combination of both measures, especially since the relationship between MIP and critical outcomes has been largely described for critically ill patients [6]. However, this requires further confirmation, and MEP could still provide relevant information when MIP cannot be measured, even though such situation would rarely occur in clinical practice.

The current observational study has provided new insights into the investigation of expiratory muscle strength in evaluating critical outcomes in critically ill patients by measuring MEP. However, this study did have some limitations. Firstly, the observational design may have induced bias. Furthermore, a convenience sample based on a primary observational study, designed for a 1-year period of inclusion, was used. Secondly, the multivariate logistic regression model power may have been limited by the low number of extubation failures (18/124 patients). Nevertheless, adjustment by principal component analysis was undertaken to account for this low occurrence rate [18]. Thirdly, the use of strategies aimed at avoiding reintubation (i.e. standard oxygen, NIV, mechanical in–exsufflation and chest physiotherapy) were not recorded in the present study. Nonetheless, each of these strategies was available and used whenever needed based on clinicians’ decisions for these patients, regardless of the study protocol. Finally, the MEP measurements were taken using measurement methods which are currently only known to be reliable for non-intubated patients; specific recommendations for intubated patients do not yet exist [13, 16]. Moreover, only maximal expiratory muscle strength data were reported; additional data, such as the quantification of expiratory muscle efforts during MV (e.g. by measuring breathing work or the activation of expiratory muscles using electromyography) or direct measurement of abdominal-wall muscle thickness (via ultrasound), would be necessary to more fully understand the impact of expiratory muscle strength on critical outcomes in mechanically ventilated patients.

## Conclusion

Higher MEP values were associated with a lower risk of extubation failure in the univariate logistic regression analyses and also in the multivariate logistic regression analyses if MIP was excluded. MEP was no longer independently associated with extubation failure when MIP was included in the model. This study was not able to differentiate the respective abilities of MEP, MIP, and their combination to discriminate patients with extubation failure or premature death. Both tools can easily be used at patients’ bedsides, but this study did not find convincing evidence that MEP alone, or the combination of both measures, was more relevant than MIP in critically ill patients. Future studies are needed to investigate all aspects of expiratory muscle function and activation during mechanical ventilation to precisely determine the role of expiratory muscles on critical outcomes.

## Data Availability

The datasets used and/or analysed during the current study are available from the corresponding author on reasonable request.

## Abbreviations

MV: Mechanical ventilation
MIP: Maximal inspiratory pressure
MEP: Maximal expiratory pressure
ICU: Intensive care unit
STROBE: Strengthening the reporting of observational studies in epidemiology
SBT: Spontaneous breathing trial
ICU-Aw: ICU-acquired weakness
ROC: Receiver operating characteristics
AUC: Area under the curve
BMI: Body mass index
VIF: Variance inflation factor
NIV: Non-invasive ventilation
